# Fast, Cost-effective and Energy Efficient Mercury Removal-Recycling Technology

**DOI:** 10.1038/s41598-018-34172-6

**Published:** 2018-11-02

**Authors:** Mainak Ganguly, Simon Dib, Parisa A. Ariya

**Affiliations:** 1 Department of Atmospheric and Oceanic Sciences, McGill University, Montreal, Quebec H3A 0B9 Canada; 2 Department of Chemistry, McGill University, Montreal, Quebec H3A 0B8 Canada

**Keywords:** Pollution remediation, Nanoparticles

## Abstract

We herein present a novel and sustainable technology for mercury recycling, with the maximum observed uptake capacity. Facile synthesis of the most efficient (~1.9 gg^−1^) nano-trap, made of montmorillonite-Fe-iron oxides, was performed to instantaneously remove mercury(II) ions from water. Elemental Hg was recovered from the adduct, by employing Fe granules, at ambient conditions. Varied pHs and elevated temperatures further enhanced this already highly efficient recycling process. The reduction of Hg(II) to Hg(I) by the nano trap and Hg(I) to Hg(0) by Fe granules are the main driving forces behind the recycling process. Facile sustainable recycling of the nano-trap and Fe granules require no additional energy. We have further developed a recyclable model for Hg nano-trap, which is inexpensive (<$5 CAD), and can remove mercury in a few seconds. This technology has multiple applications, including in the communities exposed to mercury contamination.

## Introduction

Mercury has played key roles in human lives over several thousands of years, from medicine, optics, catalysis, and more recent energy-efficient technologies^1,2^. However, in the environment, Hg is one of the most poisonous global pollutants. Hg compounds are considered to be persistent and bio-accumulative toxicants. The severe toxicity of mercury is quite compound-selective, and usage of such compounds has become a concern in the last few decades. As such, the United Nation International Treaty of Minamata was adopted in 2013 by close to 140 countries. This treaty is designed to protect human health and the environment from anthropogenic emissions of mercury containing compounds.

Developing highly efficient protocol for decontamination of water from mercury is vital for the protection of health and ecosystem. Biomass and natural bio-sorbents (namely: jute nanofibers, lichen, calcium alginate, gum karaya, chitin, chitosan, sawdust etc.), activated carbon, different types of charcoals, silica, iron oxides (of numerous phases), zero valent iron, terpolymer, aluminium oxide etc. are often used for Hg(II) removal, as mentioned in the following section. They are often treated with complex and costly molecules to design the sorbent of the practical application. We have summarised over 47 reported materials with their maximum Hg adsorption capacity.

Electrochemistry^2^ or complex redox reactions^3^ are often followed for effective recycling of Hg. In this paper, we have used iron-iron oxides for instantaneous and efficient removal of mercury from aqueous solution, and iron granules to recover elemental mercury very quickly. It is an instantaneous, cheap and efficient Hg recycling process, in contrast to most existing protocols.

## Results and Discussion

### Characterization, extraction and quantification

We synthesized montmorillonite capped iron-iron oxides (MtFe) by reducing the mixture of montmorillonite-FeCl_2_.4H_2_O with NaBH_4_ to Fe(0), and subsequently oxidising by aerial oxygen. MtFe is a black powder. The role of montmorillonite is to prevent unwanted aggregation during the reduction of iron chloride^4^. The sandwich-structure of montmorillonite, consisting of one octahedral Al–O sheet between two tetrahedral Si–O sheets, acts as a supporting matrix to disperse zero-valent iron to minimize its aggregation^5^.

When MtFe was suspended in water containing mercury(II) ion, MtFe extracted Hg immediately and quantitatively. Rate of extraction is an important issue for environmental cleanup and industrial applications. MtFe adsorbed Hg as soon as it came in contact with aqueous HgCl_2_ during sonication.

**Figure 1:**
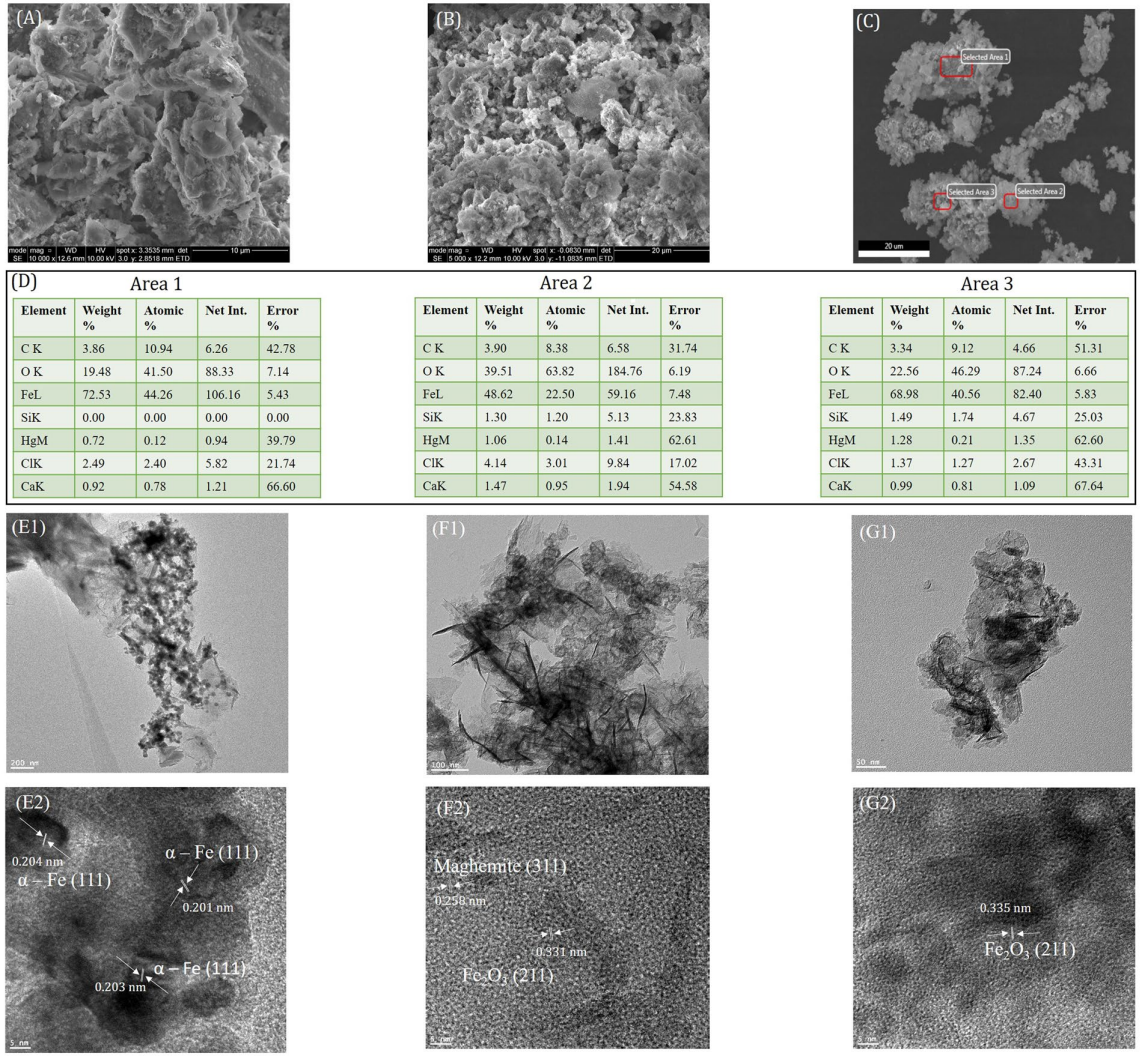
High resolution SEM images of: (**A**) MtFe, (**B**) MtFe-Hg and (**C**) Fe-MtFe-Hg; (**D**) Selected area EDAX analysis of Fe-MtFe-Hg in Fig. (**C**); (**E1**), (**F1**) and (**G1**) are the high resolution TEM images of MtFe, MtFe-Hg and Fe-MtFe-Hg; (**E2**), (**F2**) and (**G2**) are the HR-TEM images of MtFe, MtFe-Hg and Fe-MtFe-Hg.

**Figure 2:**
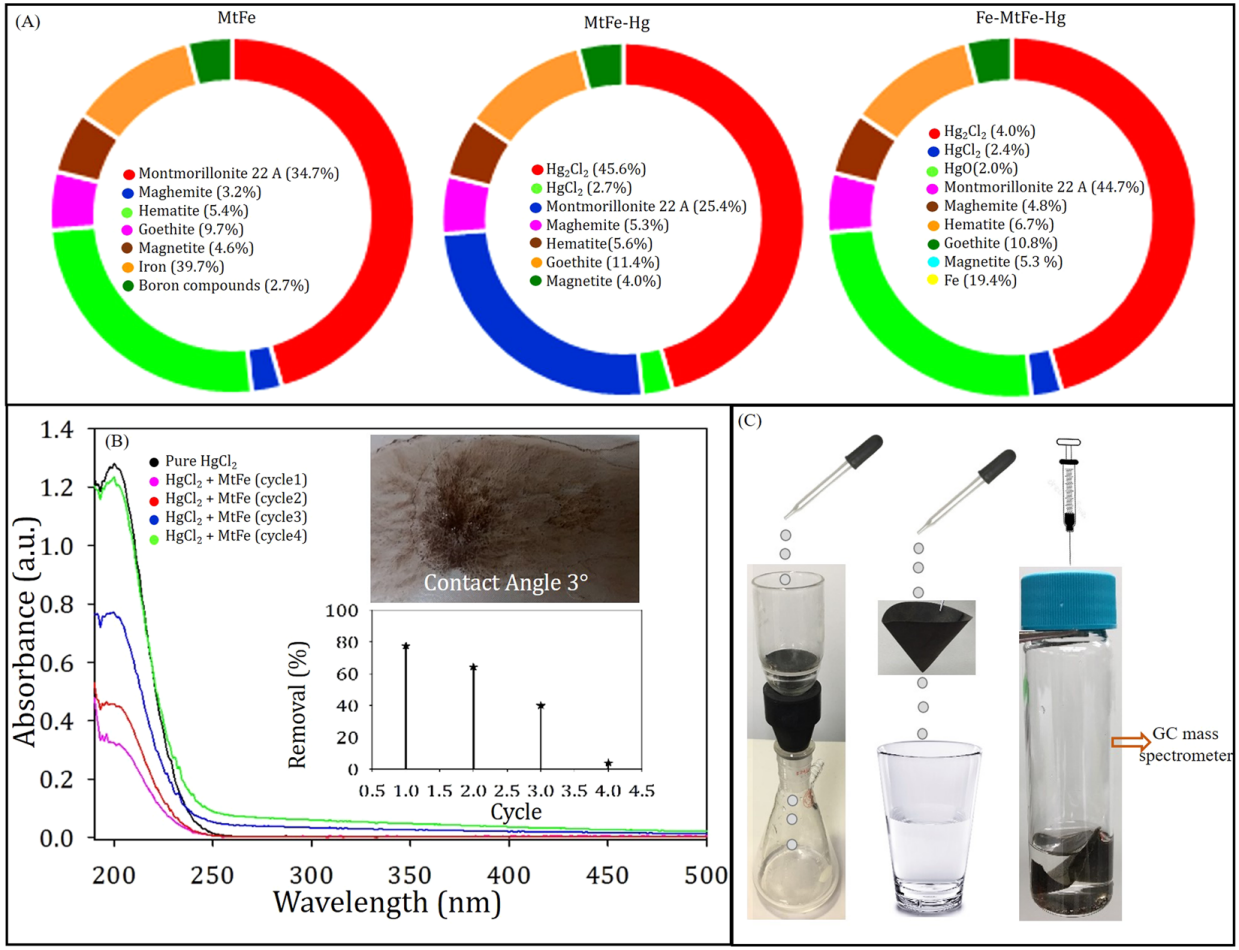
(**A**) Composition from XRD analysis of MtFe, MtFe-Hg and Fe-MtFe-Hg; (**B**) Adsorption spectra of 1 mL 10^−2^ M HgCl_2_ with 0.0025 g MtFe at different cycles and insets represent removal (%) and digital image of a 10 μL drop on the thin film of MtFe; and (**C**) MtFe impregnated sintered funnel and filter paper for decontamination of Hg contaminated water and recovery of elemental Hg from the filter paper using Fe granules.

MtFe, though insoluble in water, can easily be suspended in water due to its inorganic nano-structured layer (SEM)^6^ with hydrophilic behaviour (contact angle 6°)^7^ (Figs 1 and 2). MtFe is composed of montmorillonite 22A 34.7%, maghemite 3.2%, hematite 5.4%, goethite 9.7%, magnetite 4.6% and Fe 39.7%.

MtFe adsorbed Hg(II) to form MtFe-Hg, mainly due to electrochemical interaction. Hg(II) was converted to Hg(I). The composition of MtFe-Hg contained 45.6% Hg_2_Cl_2_, 2.7% HgCl_2_, 25.4% montmorillonite 22A, 5.3% maghemite, 5.6% hematite, 11.4% goethite and 4% magnetite (as confirmed from XRD analysis, Fig. 2A and Supplementary Figs S1–S3).

Iron was completely oxidised to adsorb Hg. The addition of Fe granules in aqueous suspension of MtFe-Hg (Fe-MtFe-Hg) quickly produced elemental Hg efficiently. Fe-MtFe-Hg contained 4% Hg_2_Cl_2_, 2.4% HgCl_2_, 2% HgO, 47.7% montmorillonite 22A, 4.8% maghemite, 6.7% hematite, 10.8% goethite and 5.3% magnetite. The lattice fringes [TEM and HRTEM]^8–10^ of MtFe, MtFe-Hg, and Fe-MtFe-Hg supported the results of XRD. SEM images indicated the rough surface morphology of MtFe-Hg and Fe-MtFe-Hg. Fe-MtFe-Hg contained insignificant Hg (EDAX) signifying the exclusive evolution of elemental Hg efficiently.

The surface of Fe granules in Fe-MtFe-Hg was hierarchical rod-like due to the oxidation for reducing Hg(I) to Hg(0). Highly enhanced white spots on the surface of Fe granules (SEM) indicated the surface oxidation and insignificant attachment of Hg to the granules (confirmed by EDAX analysis) (Supplementary Fig. S4).

### Driving force of Hg adsorption

The highly facile redox interaction was the driving force for efficient and fast adsorption of Hg(II) on MtFe. Thus, we could not recover adsorbed Hg(II) in its +2 oxidation state from MtFe-Hg [Hg adsorbed MtFe] by shaking with water/aqueous Na_2_-EDTA solution (that makes strong chelate with Hg^2+^)^11^, further indicating chemisorption type of behavior (confirmed from UV-visible spectra, Supplementary Fig. S5).

Aqueous Na_2_-EDTA treatment produced a broad band at lower energy region (~60 nm), indicating the formation of iron hydrosol and Hg(I)-EDTA complex. In brief, we were able to decontaminate aqueous solution from Hg within a few seconds using the MtFe nano-trap efficiently.

### MtFe: An exclusive nano trap of mercury

To understand the high efficiency of MtFe for Hg(II) removal, we replaced MtFe by several commercially available iron oxides (namely, hematite, magnetite and maghemite) and iron dust. We also prepared montmorillonite passivated iron nanoparticles, TEFe using green tea, as reported by KSV^12^. Neither hematite, magnetite, maghemite & TEFe nor iron dust could adsorb Hg(II), present in an aqueous system.

Furthermore, we synthesized BHFe, iron oxides, synthesized using similar protocol of MtFe, without using montmorillonite. BHFe was also able to adsorb Hg(II) from water. Yet, the efficiency per unit gram of iron is better for MtFe, as mentioned in the following section.

Montmorillonite not only enhanced the surface to volume ratio of studied iron nanoparticles, but also helped to accommodate adsorbed Hg owing to its sheet structure (two silica tetrahedral sheets with a central alumina octahedral sheet combine to produce a common layer^13^). Only montmorillonite was found to be an insignificant Hg adsorbent (Supplementary Fig. S6).

**Figure 3:**
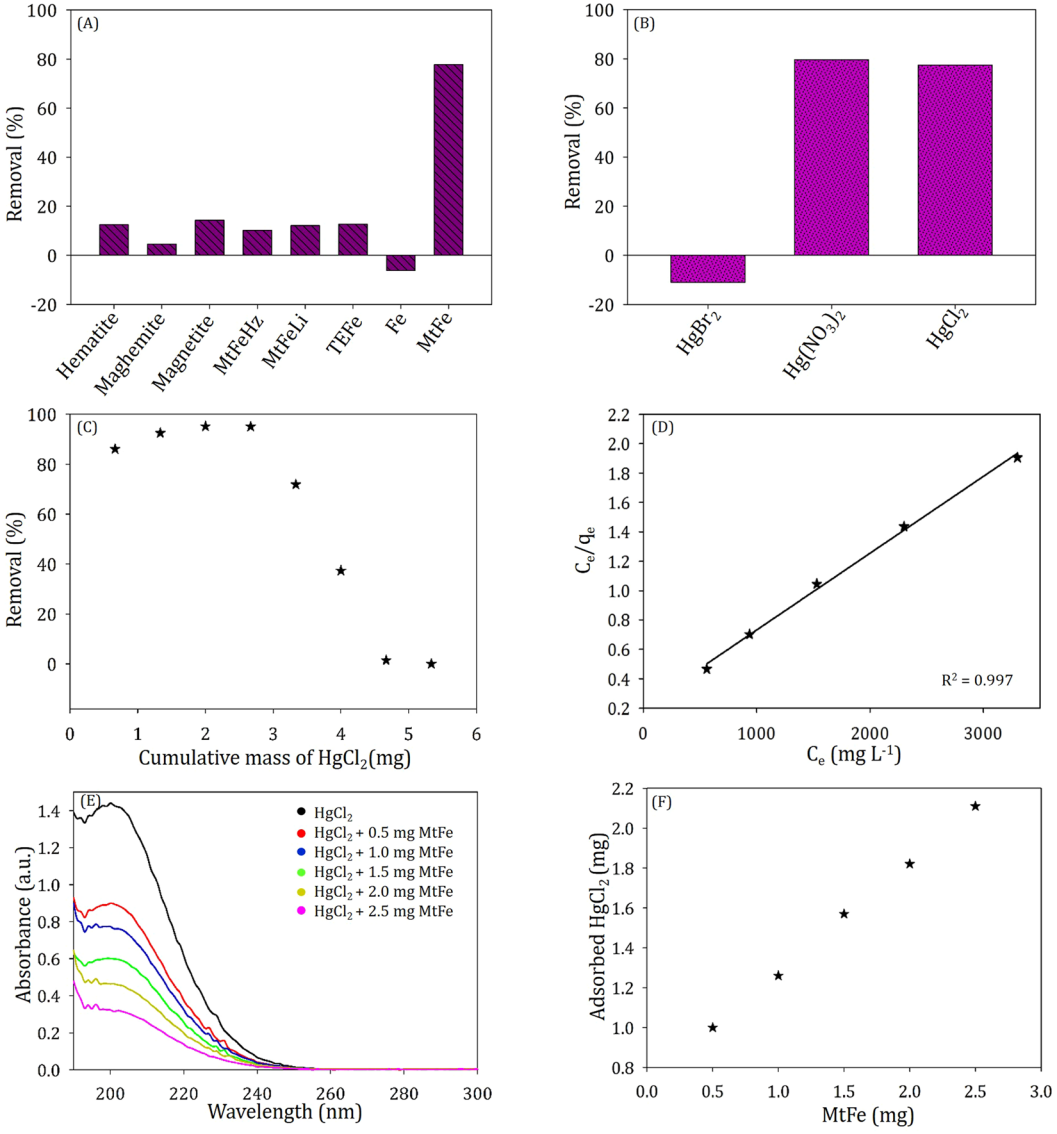
Comparison of the Hg removal efficiency of: (**A**) Different commercially available iron oxides & Fe powder with MtFe and (**B**) MtFe with different Hg(II) salt; (**C**) Removal efficiency (%) with different masses of HgCl_2_; (**D**) Langmuir adsorption plot of varied [Hg(II)] with fixed mass of MtFe; (**E**) UV-visible spectra and (**F**) Adsorbed HgCl_2_ for 1 mL 10^−2^ M HgCl_2_ with different masses of MtFe.

During synthesis, we also replaced NaBH_4_ by other well-known reducing agents namely hydrazine^14^ and LiAlH_4_^15^, and produced MtFeHz and MtFeLi. Note that for the synthesis of MtFeLi, we replaced water/ethanol with THF, due to high reactivity of water & ethanol with LiAlH_4_. MtFeHz and MtFeLi could not absorb Hg(II) similar to MtFe, and was not used in the development of this nanotechnology (Fig. 3(A) and Supplementary Fig. S7).

### Effect of counter anions on Hg adsorption

HgCl_2_ and Hg(NO_3_)_2_ could be removed by employing MtFe with equal efficiency. However, HgBr_2_ changed its chemical identity in water with some yellow precipitate. The effect of MtFe on HgBr_2_ was not understood with confidence, although peak intensity at ~230 nm (related to HgBr_2_) was decreased significantly.

HgCl_2_ was previously used as an antiseptic. It is still used as wood preservative, dry battery depolarizer, photographic intensifier, tanning agent for leather, catalyst in the chemical manufacture industry (vinyl chloride, disinfectants etc.), separating lead from gold, and others. In the felting industry, mercuric nitrate is usually used. The use HgBr_2_ is quite rare in industrial scale. Drinking water is mostly contaminated with HgCl_2_^16–18^. We can speculate that MtFe shall be quite useful as novel technology for decontamination of water (Fig. 3(B) and Supplementary Fig. S8).

### The compatibility of UV and mass spectral results

When the 4000 pg of HgCl_2_ was treated with MtFe and the supernate solution was treated with KMnO_4_ and SnCl_2_ solution^19^, the evolved Hg was estimated by GC mass spectrometry, which indicated 94.75% removal of Hg (by UV spectrometer 96.00%). The results of mass and absorption spectra, thereby, supported each other (data not shown).

### Adsorption isotherm for maximum uptake coefficient

To judge the efficiency of MtFe as a nano-trap, for Hg(II) removal, the amount of MtFe was varied with a fixed amount of HgCl_2_. The removal (%) of mercury increased monotonously with the increase of MtFe. Similarly, we gradually increased the Hg(II) concentration in steps with 2.5 mg MtFe.

We subsequently fitted our data in three adsorption isotherms, Langmuir, Freundlich & Elovich and calculated maximum adsorption capacity (Fig. 3 and Supplementary Fig. S9). The Langmuir adsorption isotherm, assuming homogenous adsorption sites and mono-layer coverage, follows the following equation^20^.

1 $$\frac{{C}_{e}}{{q}_{e}}=\frac{1}{{K}_{L}{Q}_{m}}+\frac{{C}_{e}}{{Q}_{m}}\,$$

C_e_ is the equilibrium concentration of Hg (mg L^−1^) in solution, while q_e_ is the adsorbed Hg (mg) per gram of MtFe. From the slope of the line of C_e_/q_e_ against C_e_, the maximum adsorption capacity (Q_m_) is found.

Freundlich adsorption isotherm, allowing heterogeneous adsorption sites, follows the following equation.

2 $$\mathrm{log}({q}_{e})=\,\mathrm{log}({K}_{F})+\frac{1}{n}\,\mathrm{log}({C}_{e})$$

From the slope of the line of log(q_e_) against log(C_e_), the adsorption intensity (n) and Freundlich constant (K_F_) are obtained. Q_m_ can be obtained by replacing C_e_ by C_0_ (Hg concentration in solution just after 100% adsorption).

Elovich adsorption isotherm, representing multilayer adsorption, follows the following equation.

3 $$\mathrm{ln}(\frac{{q}_{e}}{{C}_{e}})=-\,\frac{{q}_{e}}{{Q}_{m}}+\,\mathrm{ln}({K}_{E}{Q}_{m})$$

Q_m_ and Elovich adsorption isotherm constant (K_E_) were estimated from the slope and intercept, respectively, of the linear fit of ln(q_e_/C_e_) to q_e_. Q_m_ values obtained from Langmuir, Freundlich and Elovich isotherm were 1.911 gg^−1^, 1.905 gg^−1^ and 2.028 gg^−1^, respectively for HgCl_2_. However, the correlation coefficient (R^2^) of the first two was 0.997 and 0.999, respectively unlike Elovich isotherm (0.923). Thus, the nature of adsorption was of mono-layer coverage, along with heterogeneous adsorption sites.

The removal efficiency of BHFe was 12% more than MtFe, although MtFe had much higher Q_m_/g of Fe. Langmuir Q_m_ 6.048 gg^−1^ of Fe for HgCl_2_, considering unchanged mass of montmorillonite after NaBH_4_ treatment), as montmorillonite cannot at all adsorb Hg. It implies higher surface to volume ratio of MtFe via montmorillonite capping (Supplementary Fig. S6). From BET analysis, the surface areas of MtFe and BHFe are 67.0284 m²/g and 35.1889 m²/g, respectively.

### Effect of pH and temperature on Hg removal

For an adsorbent to be used for practical applications, the removal efficiency also has to be considered at versatile conditions, including different pH conditions. pH affects metal species in solution. It also alters the surface properties of adsorbents in terms of dissociation of functional groups and surface charge^21^.

The Q_m,_ mentioned above was measured at neutral pH and at 20 °C. It was found that removal efficiency (as well as Q_m_) was further increased at low pH as well as at high pH.

**Figure 4:**
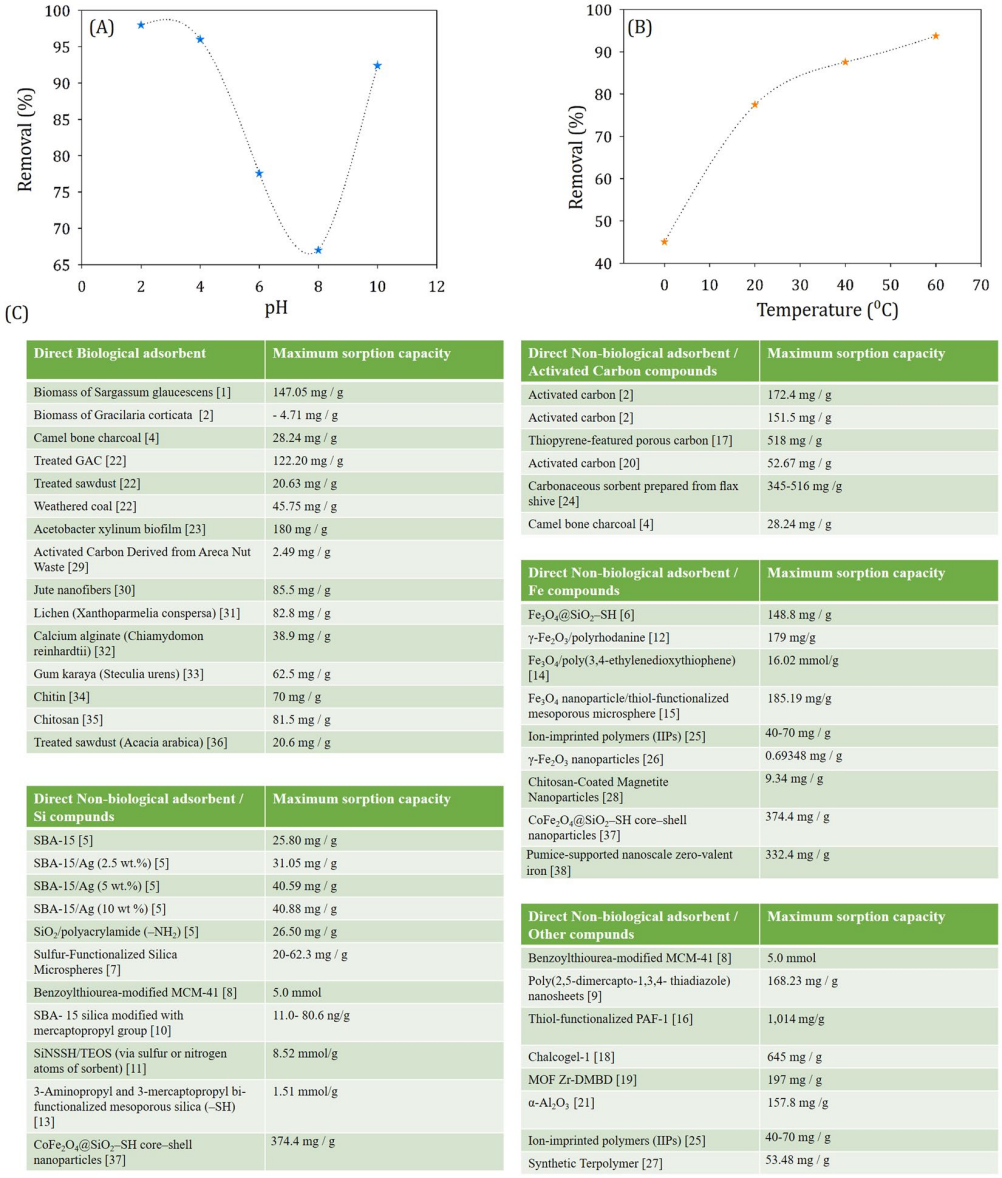
Percentage of removal of Hg with 0.0025 g of MtFe, at different (**A**) pHs and (**B**) temperatures; (**C**) Tabular representation of maximum sorption capacity of different absorbents (References are given in Supplementary Information).

We swept the pH from 2 to 10 and removal efficiencies at fixed experimental conditions were found to be 2 (98%) > 4 (96%) > 6 (77.6%) > 8 (67%) < 10 (92.4%). At lower pH, Hg^2+^ had increased binding sites for the removal of OH from the adsorbent surface, and at high pH, OH^−^ on MTFe surface attracted Hg^2+^ (Fig. 4(A) and Supplementary Fig. S10). Such pH dependent adsorption of HgCl_2_ is quite interesting in our case. Note that Zhang *et al*.^22^. reported Fe_3_O_4_@SiO_2_–SH sorbent for mercury removal and with the increase of pH, removal efficiency slowly increased.

Adsorption on Hg^2+^ was more facile at higher temperature. Removal efficiency was increased monotonously with temperature. At 60 °C, 40 °C, 20 °C and 0 °C, removal (%) of mercury was 93.8, 87.6, 77.6 and 45, respectively (Fig. 4(B) and Supplementary Fig. S11). As the process of adsorption is fast chemisorption, the adsorption capacity was enhanced due to high Brownian monition of the Hg(II), leading to effective collision with MtFe^23^.

### Recovery of Hg in +2 state

MtFe adsorbed HgCl_2_ via chemisorption with high adsorption capacity (Fig. 4(C)). It was strong adsorption. HgCl_2_ was converted to Hg_2_Cl_2_. By aging MtFe-Hg with milli-Q water for long time (5 h), we did not observe any peak of Hg(II) at 200 nm. However, we sonicated MtFe-Hg in 1 mL milli-Q water for 1 min with 10 μL concentrated HNO_3_ (strongly oxidizing) and we recovered 99.1% of adsorbed Hg(II) from HgMtFe.

### Recycling of MtFe for repeated use

MtFe-Hg was repeatedly washed with milli-Q water (via sonication and centrifugation) and then 1 mL 10^−2^ M HgCl_2_ solution was added (cycle 2). Likewise, we performed cycle 3 and cycle 4. The removal (%) for cycle 1, cycle 2, cycle 3 and cycle 4 were 77.6, 64.3, 39.9 and 3.8, respectively (Fig. 2(B)).

Vigorous washing with copious amount of water detached Hg_2_Cl_2_ from iron oxides, making room to further adsorb Hg(II) from solution. However, after four cycles, the adsorption was not practically possible. With the increase of the number of recycling cycles, Hg removal capacity of MtFe was decreased, due to permanent oxidation of Fe(0) in MtFe to Fe(III)-oxide.

### Experiments with environmental samples

We investigated removal of Hg from aqueous environmental samples and water samples from tap water (McGill University, Canada). Environmental samples included those taken from Saint Laurent river (Montreal, Canada), as well as melted snow and the rainwater, taken in downtown. Montreal^24^.

Each aqueous sample of 25 mL was examined. Various amounts of Hg (II) were added to them in order to study the removal process upon interactions with MtFe. Removal of Hg, for all the samples, was satisfactory (92–99%), under our experimental conditions, revealing the effectiveness of this method for aqueous environmental samples (Supplementary Table S1).

### Immobilization of nano trap on paper and sintered funnel

We immobilized MtFe on the Whatman filter paper and sintered glass Buchner funnel (used in gravimetric analysis^25^) (Fig. 2(D)). The Hg(II) contaminated water was passed through it and the filtered water was found to be free of mercury.

The Hg soaked filter paper or precipitate on the sintered funnel could generate elemental mercury just by adding water and Fe granules, as mentioned below.

### Recovery of elemental mercury by Fe granules

**Figure 5:**
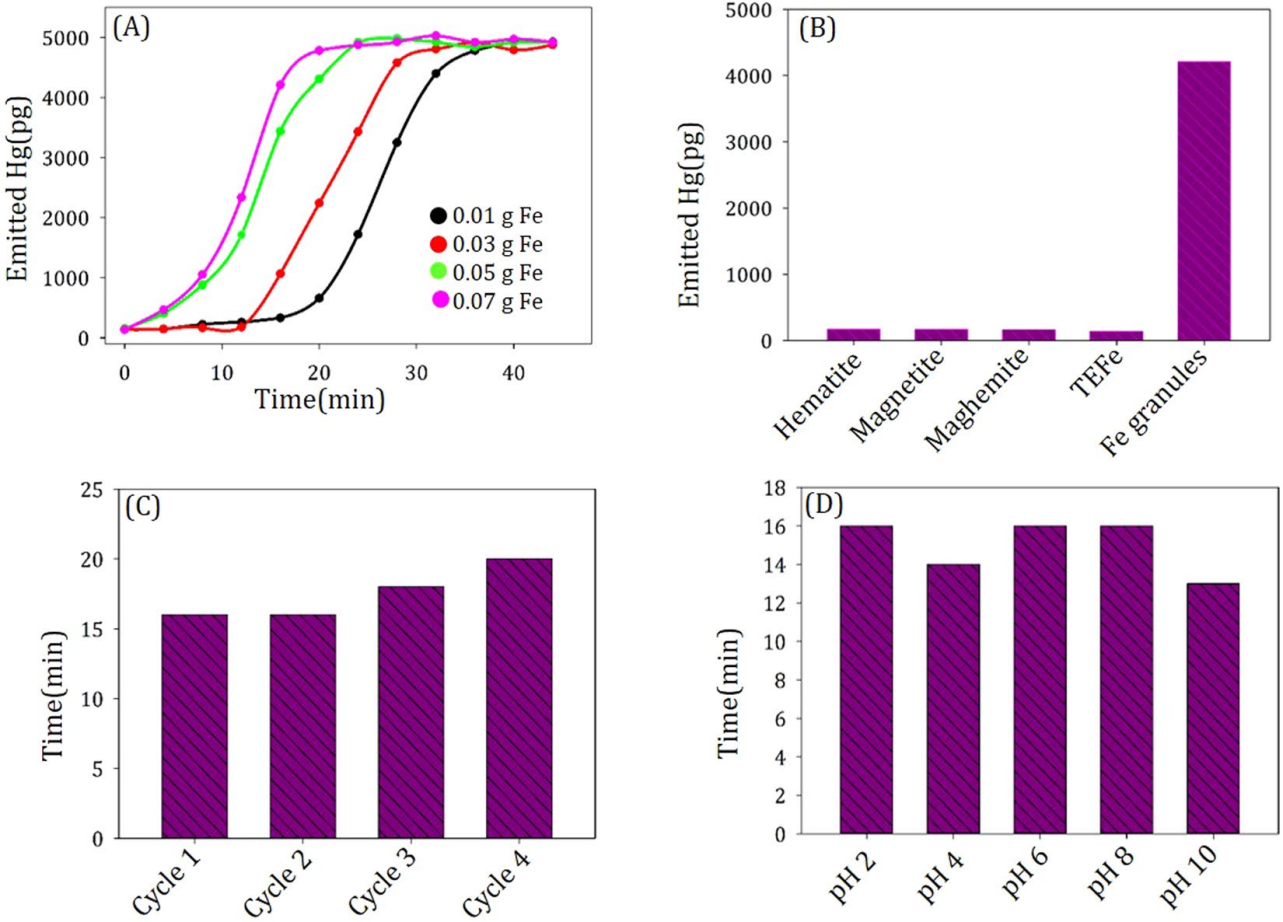
Emission of elemental Hg from MtFe-Hg, taken: (**A**) at different time intervals with Fe granules of varied masses, (**B**) with different iron compounds of 0.07 g after 16 min, (**C**) at different cycles with iron granules of 0.07 g after 16 min, and (**D**) at different pHs with iron granules of 0.07 g after 16 min.

The MtFe-Hg solution (obtained by adding 0.0020 g MtFe in 1 mL 10^−2^ M HgCl_2_) was kept in a 40 mL EPA vial (without any further treatment) and 0.07 g of Fe(0) granules was added in it. The solution was then put on an automatic shaker and evolved Hg gas (confirmed from isotopic distribution of mass spectral data), generated inside the EPA vials, was measured using a GC mass spectrometer (Fig. 5).

The Hg evolution was very fast. Within 16 min, the saturated vapor pressure of Hg (2.031558 × 10^−7^ MPa at 295.15 K)^26^ was reached (with mass ~5000 pg in the flask).

The rate of Hg evolution is directly proportional to the mass of employed Fe granules. Saturated vapor pressure was obtained after 20 min, 23 min and 31 min, when the employed Fe granules were 0.05 g, 0.03 g, 0.01 g, respectively. 0.07 g of TEFe as well as different commercially available iron oxides (hematite, magnetite, maghemite) could not produce elemental Hg in 16 minutes under similar experimental conditions, unlike the Fe granules (Fig. 5 (A,B)).

MtFe adsorbed substantial amount of Hg, during the evolution of Hg from MtFe-Hg, by Fe granules. Adsorption of elemental Hg by iron oxide is well known^20^. We have used large Fe granules (~0.01 g each) for easy separation from the reaction mixture to recycle the Fe granules.

As the surface of the Fe(0) granules usually contain iron oxides due to aerial oxidation^27^, Fe granules (that adsorbed elemental Hg) were kept overnight at 40 °C in a vacuum oven after being washed with milli-Q water to remove adsorbed Hg before performing cycle 2, 3 and 4 to eliminate any confusion.

The rate of removal of elemental Hg was almost unchanged for different cycles with the Fe granules, qualifying the criteria of the cheapest Hg recycling agent (Fig. 5(C)). Moreover, pH did not significantly influence the elemental Hg evolution. Saturated vapour pressure of Hg was obtained at 16 min, 14 min, 16 min, 16 min and 13 min, when the pH of the solution was 2, 4, 6, 8 and 10, respectively (Fig. 5(D)).

XRD spectra depicted that Hg(II) was converted to Hg(I) in MtFe-Hg. Such conversion actually helped for fast recycling of elemental mercury in our case. We treated Fe granules with HgCl_2_ and Hg_2_Cl_2_ separately and found that Hg_2_Cl_2_ could generate ~5000 pg elemental Hg vapor (a fixed value was reached in mass spectrometer due to the saturation of elemental Hg in the container) within 16 min (Supplementary Fig. S12), unlike HgCl_2_.

Formal reduction potential^28^ allows iron(0) to be oxidized to iron(III), reducing Hg(I) to elemental mercury. The control experiment of Hg_2_Cl_2_ explains the mechanism of Hg(0) production via MtFe with Fe granules. Importantly, the Fe in MtFe could not generate elemental Hg due to passivation of Fe with iron oxides and clay material, such as montmorillonite, under our experimental conditions.

### Advantage of the boron compounds in the filtrate during synthesis of MtFe

During the reduction by NaBH_4_, NaBO_2_ is usually produced^29^. The filtrate during the synthesis of MtFe is not a useless by-product. We can also use such boron compounds for the confirmation of the alcohol in water.

Boric acid ester, which forms between the reaction of boron compounds and alcohol, burn with a green flame. It indicates a potential application of boron compounds in flame photometric study and pyrotechnics for both military and civilian fireworks^30–32^.

**Figure 6:**
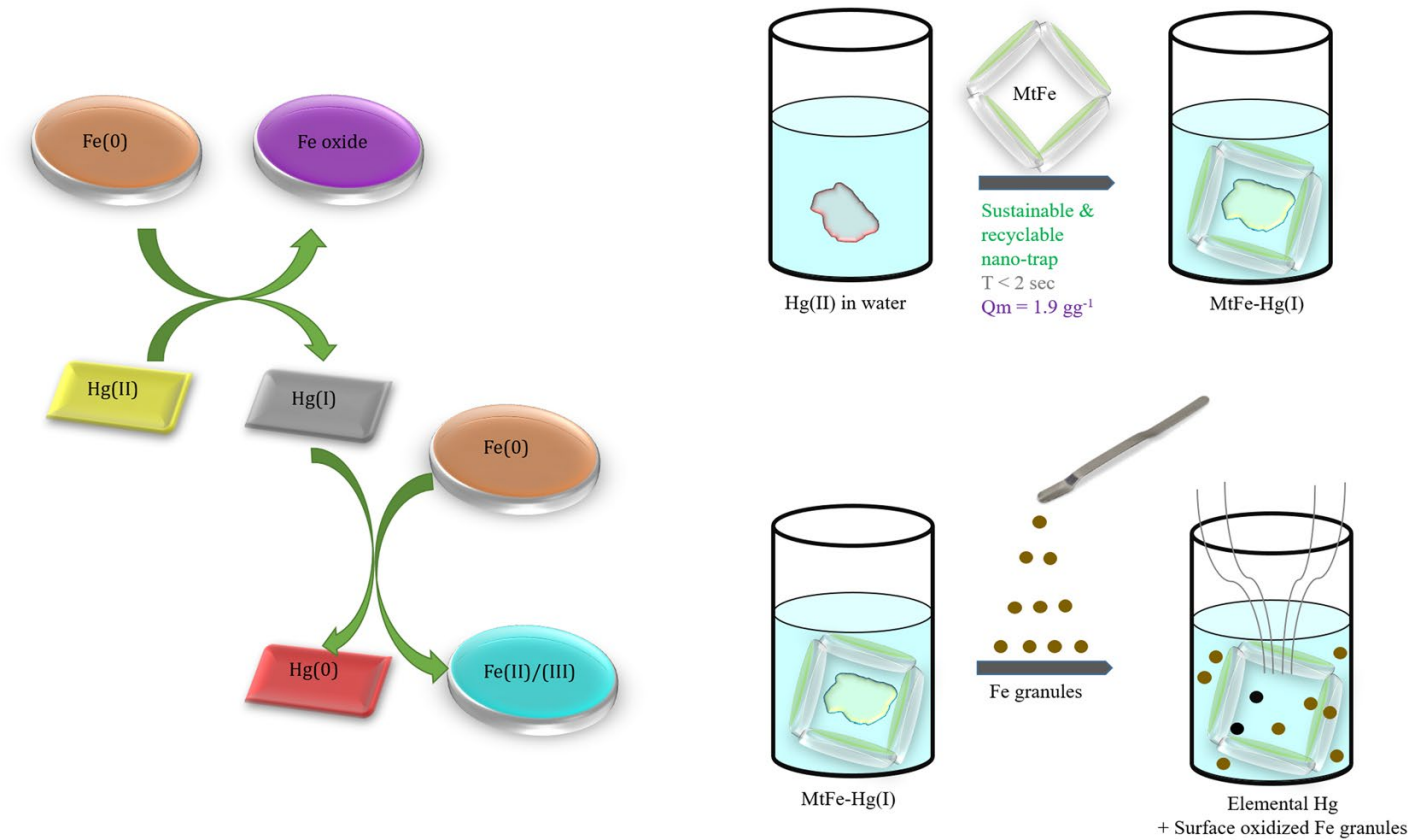
Schematic representation of recycled Hg by employing MtFe and Fe granules.

Fe(0) in MtFe was oxidized to Fe-oxide, by reducing Hg(II) to Hg(I). Employed iron granules [Fe(0)], in the solution containing Hg(I), were oxidized to Fe(II)/Fe(III), while Hg(I) was reduced to elemental Hg(0) (Fig. 6).

An instantaneous and efficient nano-trap technique has been designed to remove Hg(II) from water and recovery of elemental mercury by Fe granules by simple redox reactions (Fig. 6). The nano-trap and Fe granules are recyclable.

Our protocols are applicable to different experimental conditions (pHs, temperatures etc.) indicating the versatility of this technique. Artisanal and small-scale mining, including gold, leads to a sever mercury contamination. The developed model nano-trap impregnated sintered funnel, and filter paper for water decontamination may be a valuable kit for small-scale use.

As Fe/iron oxides/montmorillonite are natural materials, and major components of atmospheric mineral dust, our Hg recycling protocol can also be used for waste management of this toxic compound.

## Methods

### Synthesis

0.026 moles of FeCl_2_.4H_2_O were fully dissolved in a mixture of 48 mL ethanol and 12 mL water. To it, 3 g of montmorillonite was added and sonicated for 2 h to obtain a muddy brown mixture (X). 1M NaBH_4_ solution was prepared by dissolving 6.1 g of solid in 200 mL of ice-cold milli-Q water. The newly prepared NaBH_4_ solution was added drop-wise into X, showing an instant change of color from brown to black.

After the addition of the entire NaBH_4_ solution, the reaction mixture was shaken for 10 min and aged for ~7 h. The supernate was subsequently removed and the solid black precipitate was washed thoroughly with milli-Q water once and twice with 200 mL of ethanol at the end. The synthesized iron-montmorillonite composite was aged at 60 °C to dry completely. The dry black solid was crushed with a mortar to obtain fine powder MtFe.

In the X solution, Whatman 1 filter paper was dipped during sonication and then dried and dipped in NaBH_4_ solution and finally dried to obtain black MtFe impregnated filter paper. Water suspension of MtFe was filtered by glass sintered funnel to obtain MtFe impregnated funnel.

### Analytical Techniques for Physical and Chemical Characterization

#### UV-Vis spectroscopy

All UV–Vis absorption spectra were recorded from a VARIAN CARY 50 Bio UV spectrophotometer. The solutions were put into a 1 cm, well-stoppered quartz cuvette to record after base-line corrections with milli-Q water. Absorption spectra were measured after dilution of supernate for 31 times of each sample.

4 $$\mathrm{Removal}, \% =100\times ({{\rm{C}}}_{0}-{{\rm{C}}}_{{\rm{e}}})/{{\rm{C}}}_{0}$$

where C_0_ and C_e_ are the initial and equilibrium concentrations of Hg^2+^ in a solution, respectively^16^. C_0_ and C_e_ can be replaced by A_0_ (initial absorbance) and A_e_ (equilibrium absorbance), when removal was measured via UV-visible spectroscopy.

#### High resolution Transmission Electron Microscopy (TEM)

Droplets of 10 μL of MtFe (0.0025 g in 10 mL of milli-Q water), MtFe-Hg (0.0025 g MtFe in 1 mL of 10^−2^ M HgCl_2_) and Fe-MtFe-Hg (0.0025 g MtFe in 1 mL of 10^−2^ M HgCl_2_ and 0.05 g Fe granules) were deposited on glow-discharged carbon film coated copper electron microscopy grids. The droplets were kept on the grids for 1 day to dry on it. Then the samples were imaged using a FEI Tecnai 12 Biotwin TEM microscope (FEI Electron Optics) equipped with a tungsten filament at 120 kV, containing an AMT XR80C CCD Camera System.

#### High resolution Scanning Electron Microscopy (SEM)

Particle morphology was examined using a FEI Helios Nanolab 660 Dual Beam (Focused Ion Beam) Extreme High-Resolution Scanning Electron Microscope. The microscope contains Leica Microsystems EM VCT100 Cryo-Transfer System, MultiChem Gas Injection System, and EDAX Octane Ultra 100 mm^2^ SDD and TEAM 3D EDS Analysis System. Samples of MtFe, MtFe-Hg and Fe-KaFe-Hg [as mentioned in TEM analysis] were vacuum dried for 24 hours at room temperature.

#### X-ray Diffraction (XRD) Spectroscopy

X-Ray diffraction was performed with a Bruker D8 Discovery X-Ray Diffractometer (VANTEC Detector Cu-Source λ = 1.5418 Å). XRD patterns were recorded for 3° ≤ 2θ ≤ 80° with increments of 0.005°. Samples of MtFe, MtFe-Hg and Fe-KaFe-Hg [as mentioned in TEM analysis] were vacuum dried for 24 hours before measurement.

## Electronic supplementary material

Supplementary Information

## References

[1] Ariya PA (2015). Mercury physicochemical and biogeochemical transformation in the atmosphere and at atmospheric interfaces: a review and future directions. Chem. Rev..

[2] Hu Z (2016). Development of a Green Technology for Mercury Recycling from Spent Compact Fluorescent Lamps Using Iron Oxides Nanoparticles and Electrochemistry. ACS Sustainable Chem. Eng..

[3] Gash AE (1998). Efficient Recovery of Elemental Mercury from Hg(II)-Contaminated Aqueous Media Using a Redox-Recyclable Ion-Exchange Material. Environ. Sci. Technol..

[4] Ganguly M, Dib S, Ariya P (2018). Purely Inorganic Highly Efficient Ice Nucleating Particle. ACS Omega.

[5] Wu L, Liao L, Lv G, Qin F (2015). Stability and pH-independence of nano-zero-valent iron intercalated montmorillonite and its application on Cr(VI) removal. Journal of Contaminant Hydrology.

[6] Wu S (2008). A Biomimetic Hierarchical Scaffold: Natural Growth of Nanotitanates on Three-Dimensional Microporous Ti-Based Metals. Nano Lett..

[7] Yuan, Y. & Lee, T. R. Contact Angle and Wetting Properties. In *Surface Science Techniques*, Bracco, G.; Holst, B., Eds Springer: Berlin, Heidelberg, Vol. 51, pp 3–34 (2013).

[8] Kohout T (2014). Space weathering simulations through controlled growth of iron nanoparticles on olivine. Icarus.

[9] Cao DR (2016). High saturation magnetization of γ-Fe_2_O_3_ nanoparticles by a facile one-step synthesis approach. Sci. Rep..

[10] Guo Z (2007). Preparation of PVA/Co/Ag film and evaluation of its magnetic and microstructural properties. Proc. SPIE.

[11] Ganguly M (2014). Fluorescent Au(I)@Ag_2_/Ag_3_ giant cluster for selective sensing of mercury(II) ion. Dalton Trans..

[12] Gottimukkala KSV (2017). Green Synthesis of Iron Nanoparticles Using Green Tea leaves Extract. J. Nanomedicine Biotherapeutic Discov..

[13] Bhattacharyya KG, Gupta SS (2008). Adsorption of a few heavy metals on natural and modified kaolinite and montmorillonite: A review. Advances in Colloid and Interface Science.

[14] Klačanová K (2013). Formation of Fe(0)-Nanoparticles via Reduction of Fe(II) Compounds by Amino Acids and Their Subsequent Oxidation to Iron Oxides. J. Chem..

[15] Schaeffer GW, Roscoe JS, Stewart AC (1956). The Reduction of Iron (111) Chloride with Lithium Aluminohydride and Lithium Borohydride: Iron(I1) Borohydride. J. Am. Chem. Soc..

[16] Rahbar N, Jahangiri A, Boumi S, Khodayar MJ (2014). Mercury Removal From Aqueous Solutions With Chitosan-Coated Magnetite Nanoparticles Optimized Using the Box-Behnken Design. Jundishapur J. Nat. Pharm. Prod..

[17] Broussard LA, Hammett-Stabler CA, Winecker RE, Ropero-Miller JD (2002). The toxicology of mercury. Lab Med..

[18] Boujbiha MA (2012). Hematotoxicity and genotoxicity of mercuric chloride following sub-chronic exposure through drinking water in male rats. Biol. Trace Elem. Res..

[19] Camera AS (2015). Total Mercury Determination in Petroleum Green Coke and Oily Sludge Samples by Cold Vapor Atomic Fluorescence Spectrometry. J. Braz. Chem. Soc..

[20] Kurien U, Hu Z, Lee H, Dastoor AP, Ariya PA (2017). Radiation enhanced uptake of Hg0 (g) on iron (oxyhydr)oxide nanoparticles. RSC Adv..

[21] Loosli F, Stoll S (2017). Effect of surfactants, pH and water hardness on the surface properties and agglomeration behavior of engineered TiO_2_ nanoparticles. Environ. Sci.: Nano.

[22] Zhang D, Yin Y, Liu J (2017). Removal of Hg^2+^ and methylmercury in waters by functionalized multi-walled carbon nanotubes: adsorption behavior and the impacts of some environmentally relevant factors. Chemical Speciation & Bioavailability.

[23] Ganguly M, Mondal C, Jana J, Pal A, Pal T (2014). Selective Dopamine Chemosensing Using Silver-Enhanced Fluorescence. Langmuir.

[24] Rangel-Alvarado RB, Nazarenko Y, Ariya PA (2015). Snow-borne nanosized particles: Abundance, distribution, composition, and significance in ice nucleation processes. J. Geophys. Res.: Atmos..

[25] Morris AGC (1965). The volumetric determination of silica and its application to ferromanganese slag and silicomanganese analysis. Analyst.

[26] Huber ML, Laesecke A, Friend DG (2006). Correlation for the Vapor Pressure of Mercury. Ind. Eng. Chem. Res..

[27] Zhanga G, Liao Y, Baker I (2010). Surface engineering of core/shell iron/iron oxide nanoparticles from microemulsions for hyperthermia. Mater Sci. Eng. C Mater. Biol. Appl..

[28] Schilt AA (1963). Formal Oxidation-Reduction Potentials and Indicator Characteristics of Some Cyanide and 2,2/-Bipyridine Complexes of Iron, Ruthenium, and Osmium. Anal. Chem..

[29] Ouyang L, Zhong H, Li H–W, Zhu M (2018). A Recycling Hydrogen Supply System of NaBH_4_ Based on a Facile Regeneration Process: A Review. Inorganics.

[30] Steinhauser G, Klaptke TM (2008). Green” Pyrotechnics: A Chemists’ Challenge. Angew. Chem. Int. Ed..

[31] Dean JA, Thompson C (1955). Flame Photometric Study of Boron. Anal. Chem..

[32] Sabatini JJ, Poret JC, Broad RN (2011). Boron Carbide as a Barium‐Free Green Light Emitter and Burn‐Rate Modifier in Pyrotechnics. Angew. Chem. Int. Ed..

