# Author Correction: Fast, Cost-effective and Energy Efficient Mercury Removal-Recycling Technology

**DOI:** 10.1038/s41598-019-43991-0

**Published:** 2019-05-16

**Authors:** Mainak Ganguly, Simon Dib, Parisa A. Ariya

**Affiliations:** 1 Department of Atmospheric and Oceanic Sciences, McGill University, Montreal, Quebec H3A 0B9 Canada; 2 Department of Chemistry, McGill University, Montreal, Quebec H3A 0B8 Canada

Correction to: *Scientific Reports* 10.1038/s41598-018-34172-6, published online 02 November 2018

The Author Contributions section in this Article is incorrect.

“M.G. did all necessary experimental work (except Fig. 2), wrote main manuscript text and prepared all figures. S.D. did experimental work for Fig. 2. P.A.A. wrote the original proposal which was funded, and then served as the basis of this research project, supervised the work and modified the manuscript for important intellectual content.”

should read:

“M.G. did all necessary experimental work (except Fig. 2), wrote main manuscript text and prepared all figures. S.D. was an undergraduate researcher who was predominantly in charge of work shown in Figure 2, and was involved from the beginning of this project including all repetitions of the work performed by M.G. P.A.A. wrote the original proposal which was funded, and then served as the basis of this research project, supervised the work and modified the manuscript for important intellectual content.”

