# Supplementary material for: Fast, Cost-effective and Energy Efficient Mercury Removal-Recycling Technology
Source: Sci Rep. 2018 Nov 2;8:16255. doi: 10.1038/s41598-018-34172-6 (PMC6214898; doi:10.1038/s41598-018-34172-6)
Supplement: Supplementary file 1 — Supplementary Information [file 41598_2018_34172_MOESM1_ESM.docx]

**Supplementary Information**

**Fast, Cost-effective and Energy Efficient Mercury Removal-Recycling Technology**

Mainak Ganguly,^a^ Simon Dib^b^ and Parisa A. Ariya*^a,b^

^a^Department of Atmospheric and Oceanic Sciences, McGill University, Montreal, Quebec H3A 0B9, Canada;  ^b^Department of Chemistry, McGill University, Montreal, Quebec H3A 0B8, Canada


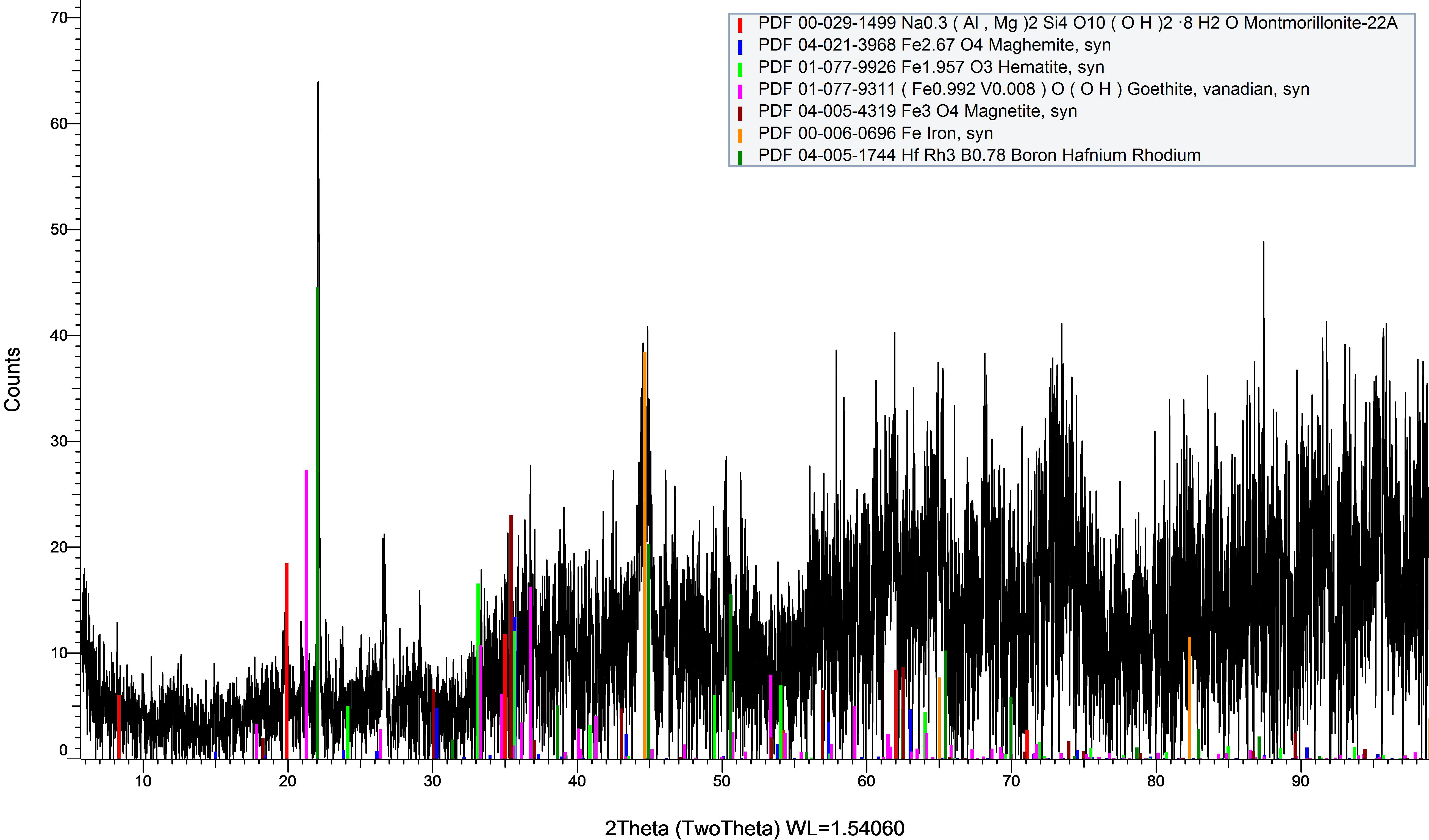


**Figure S1: The XRD spectrum of MtFe with software matching.**


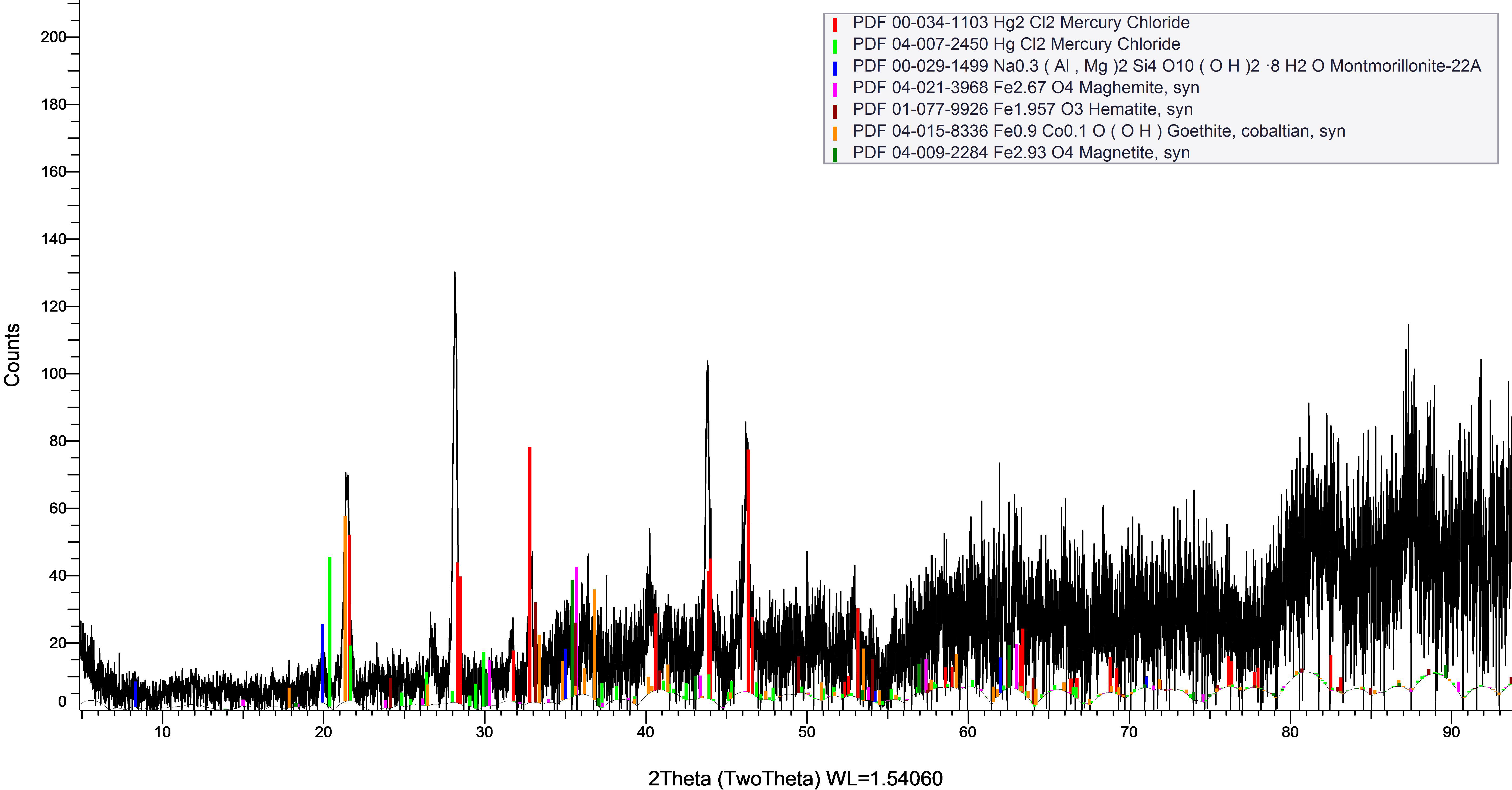


**Figure S2: The XRD spectrum of MtFe-Hg with software matching.**


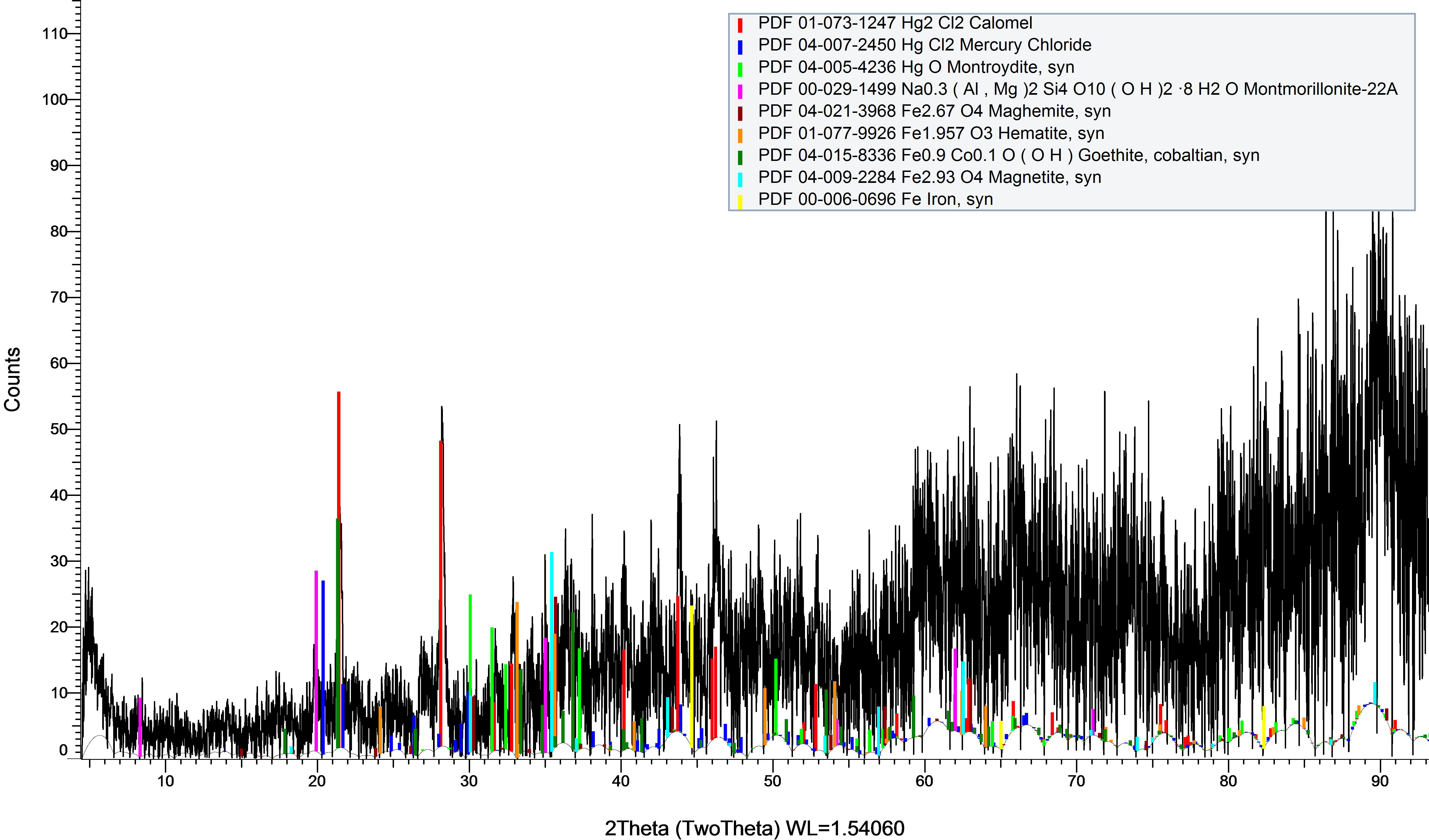


**Figure S3: The XRD spectrum of Fe-MtFe-Hg with software matching.**


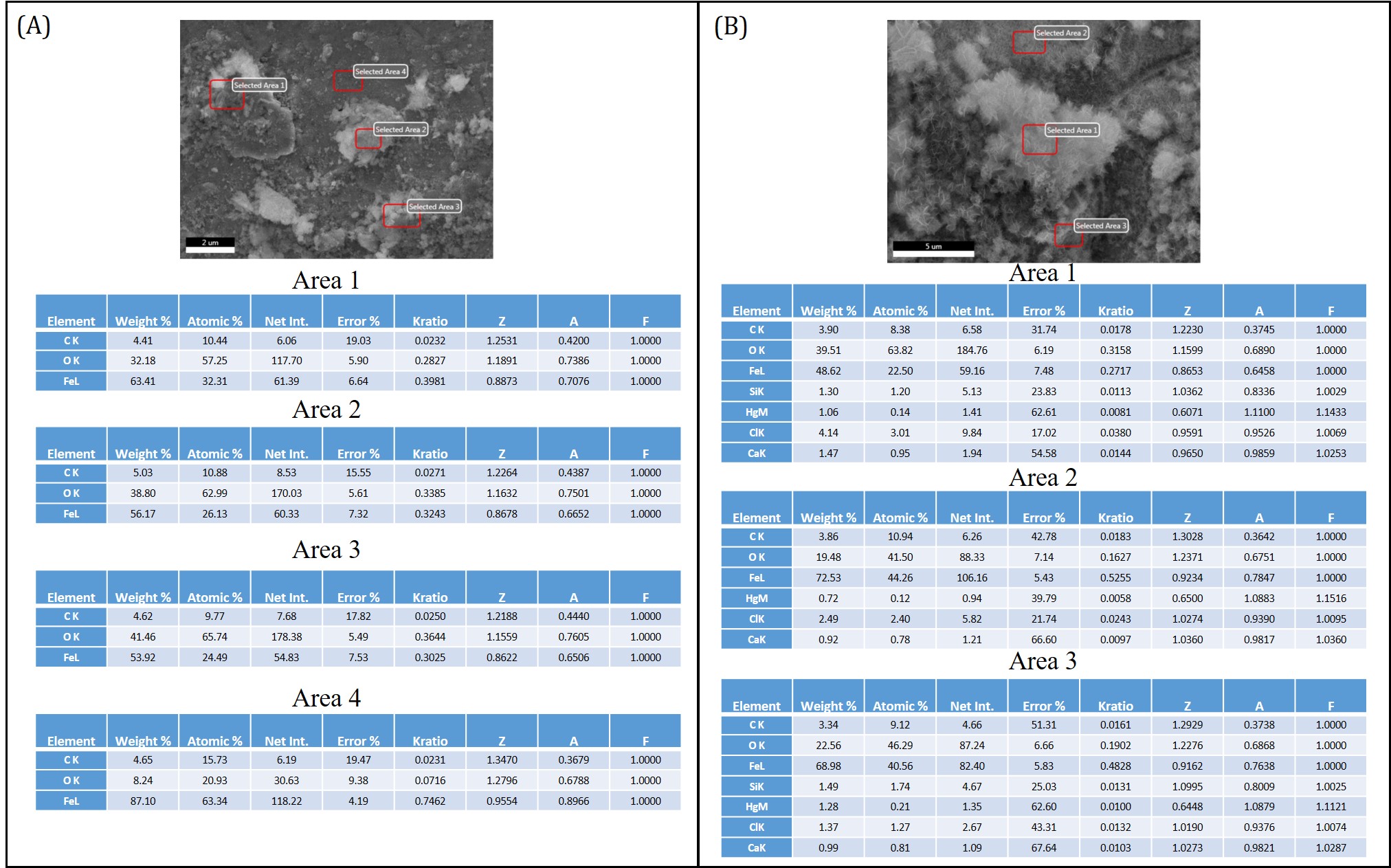


**Figure S4: SEM image and selected area EDAX analysis of Fe granules (A) before and (B) after the reaction with MtFe-Hg.**


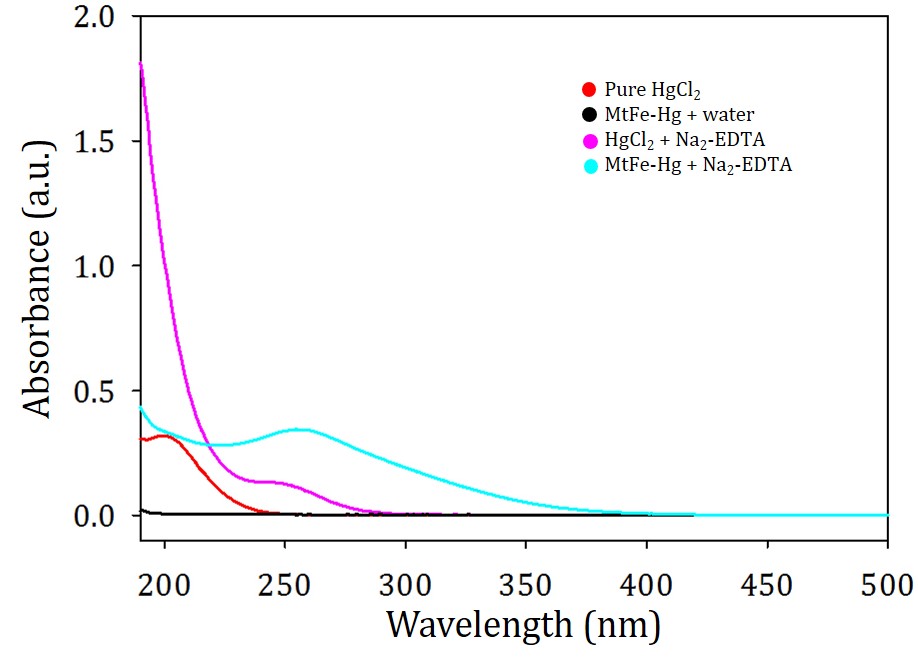


**Figure S5: UV-vis spectra of pure HgCl_2_, MtFe-Hg precipitate after shaken with water (centrifuged and supernate was collected), 1:1 complex of Na_2_EDTA - HgCl_2_ and MtFe-Hg precipitate after shaken with Na_2_EDTA solution (centrifuged and supernate was collected). MtFe-Hg precipitate was obtained by adding 0.0025 g MtFe in 1 mL 10^-2^ M HgCl_2_. Then, Na_2_EDTA was added at 1:1 ratio. Then, solution was 120 times diluted before measuring absorption spectra.**


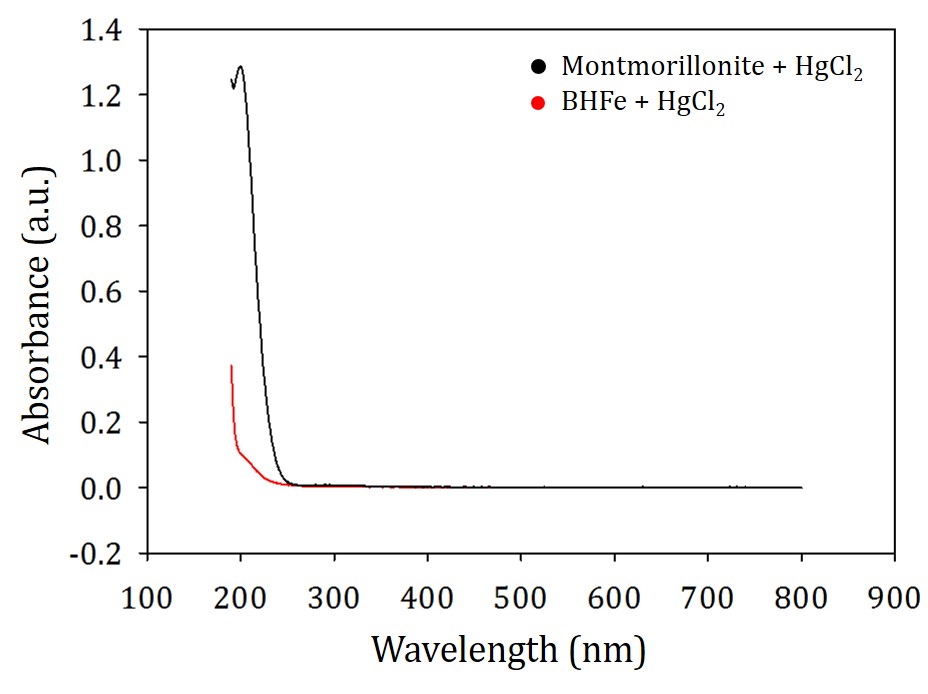


**Figure S6: UV-vis spectra of pure HgCl_2_ + Montmorillonite and HgCl_2_ + BHFe. Montmorillonite and BHFe of 0.0025 g was added in 1 mL 10^-2^ M Hg(II) solution and sonicated. Then, supernate solutions were 31 times diluted before measuring absorption spectra.**


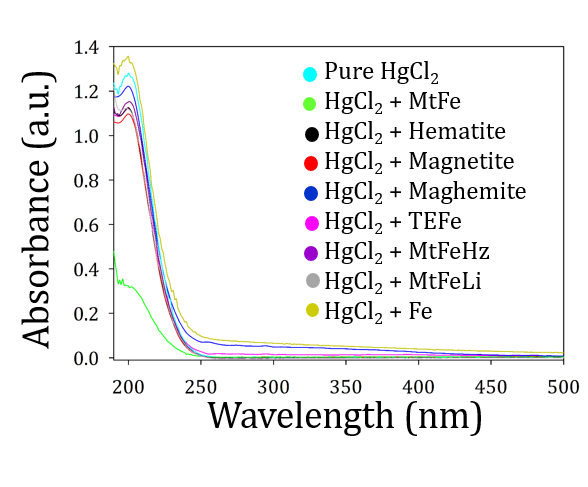


**Figure S7: UV-vis spectra of pure HgCl_2_ and HgCl_2_ with different iron oxides. Iron oxides of 0.0025 g was added in 1 mL 10^-2^ M Hg(II) solution. Then, supernate solutions were 31 times diluted before measuring absorption spectra.**


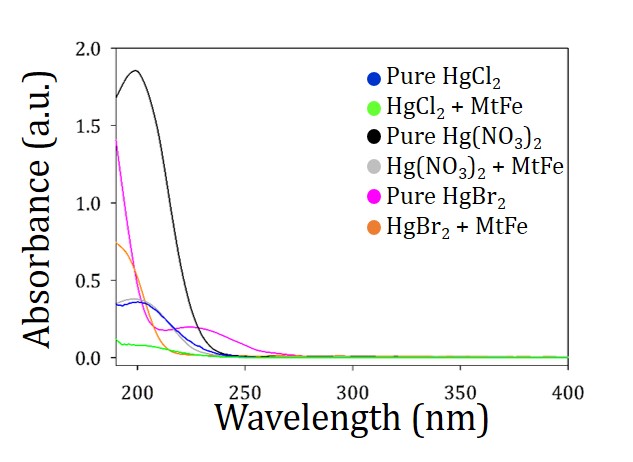


**Figure S8: UV-vis spectra of** **pure** **HgCl_2_,** **HgCl_2_ + MtFe, pure** **Hg(NO_3_)_2_, Hg(NO_3_)_2_ + MtFe, pure HgBr_2_ and HgBr_2_ + MtFe. MtFe of 0.0025 g was added in 1 mL 3˟10^-3^ M Hg(II) solution. Then, supernate solution was 31 times diluted before measuring absorption spectra.**


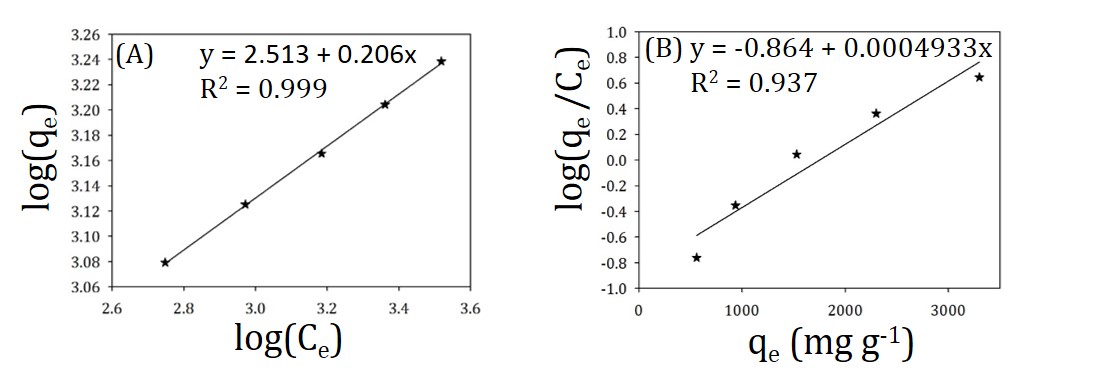


**Figure S9: (A) Freundlich plot of Hg adsorption on MtFe; (B) Elovich plot of Hg adsorption on MtFe.**


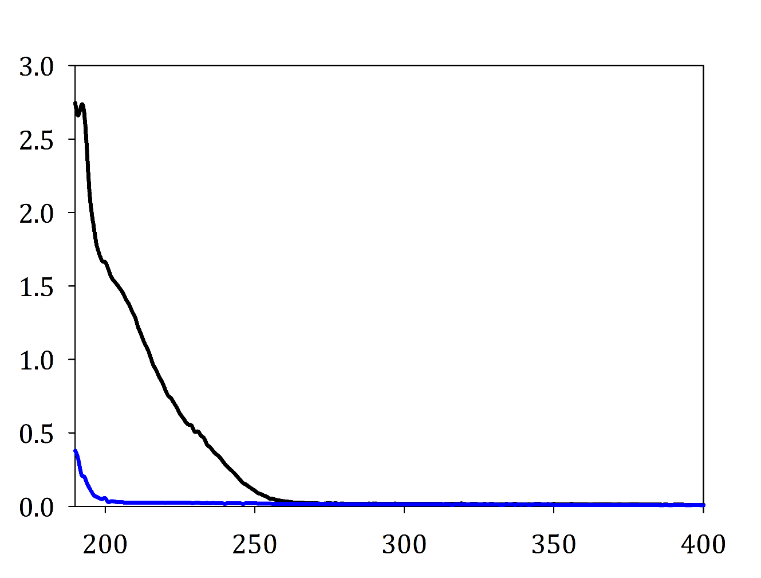


Absorbance (a.u.)

Wavelength (nm)


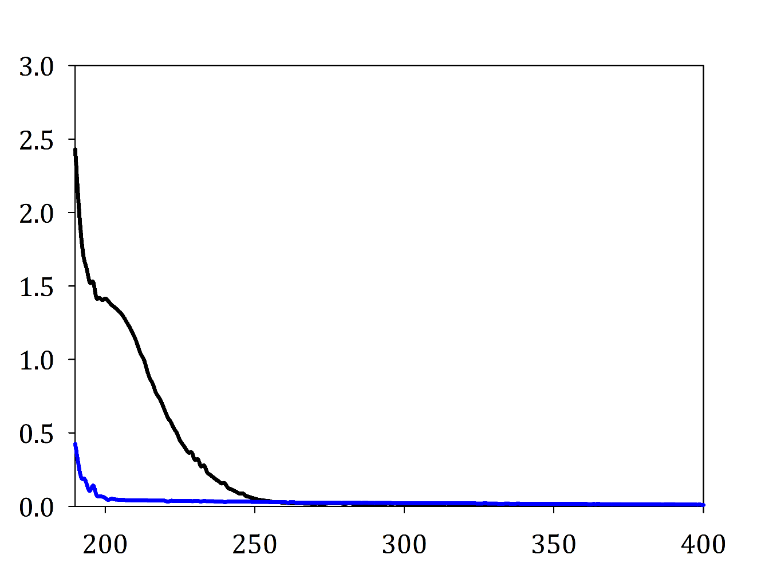


Absorbance (a.u.)

Wavelength (nm)

HgCl_2_ (at pH 4)

MTFe + HgCl_2_ (at pH 4)

HgCl_2_ (at pH 2)

MTFe + HgCl_2_ (at pH 2)


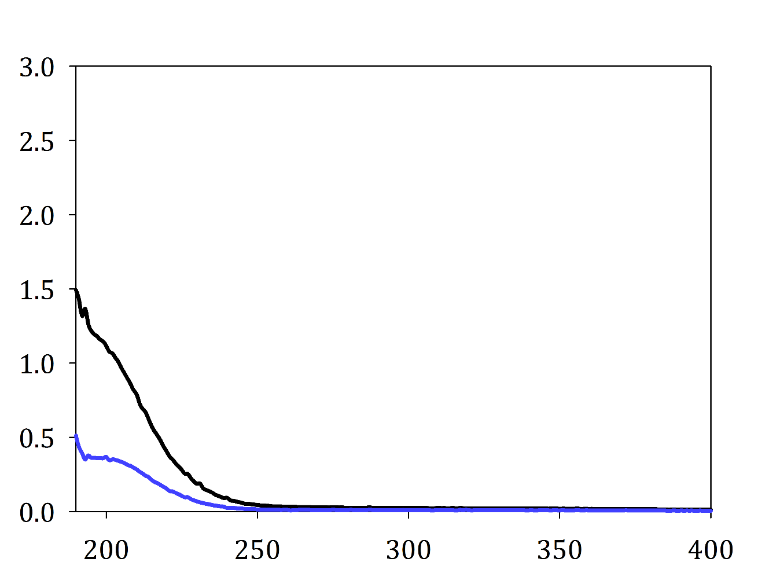


Absorbance (a.u.)

Wavelength (nm)

HgCl_2_ (at pH 8)

MTFe + HgCl_2_ (at pH 8)


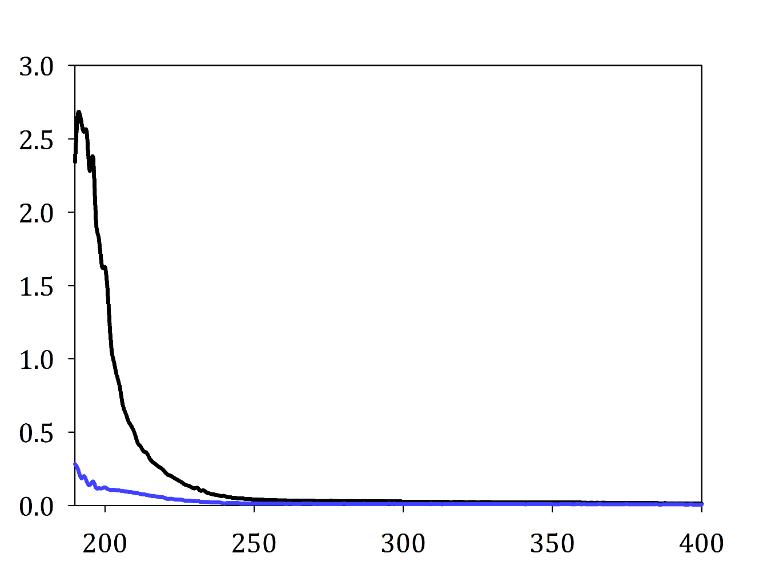


Absorbance (a.u.)

Wavelength (nm)

HgCl_2_ (at pH 10)

MTFe + HgCl_2_ (at pH 10)

**Figure S10: UV-vis spectra of pure HgCl_2_ + MtFe, at different pHs. MtFe of 0.0025 g was added in 1 mL 10^-2^ M Hg(II) solution and sonicated. Then, supernate solution was 31 times diluted before measuring absorption spectra.**


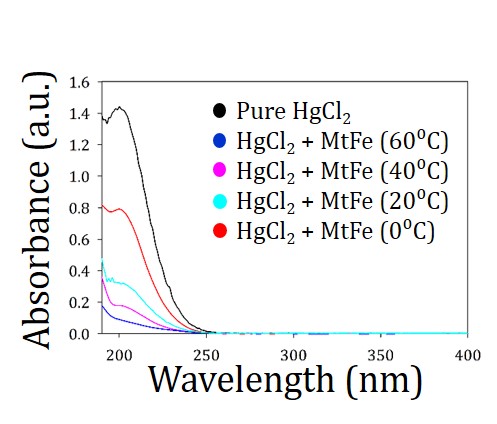


**Figure S11: UV-vis spectra of pure HgCl_2_ and (HgCl_2_ + MtFe) at different temperatures. MtFe of 0.0025 g was added in 1 mL 10^-2^ M Hg(II) solution and sonicated at different temperatures and centrifuged. Then, supernate solutions were diluted 31 times before measuring absorption spectra.**


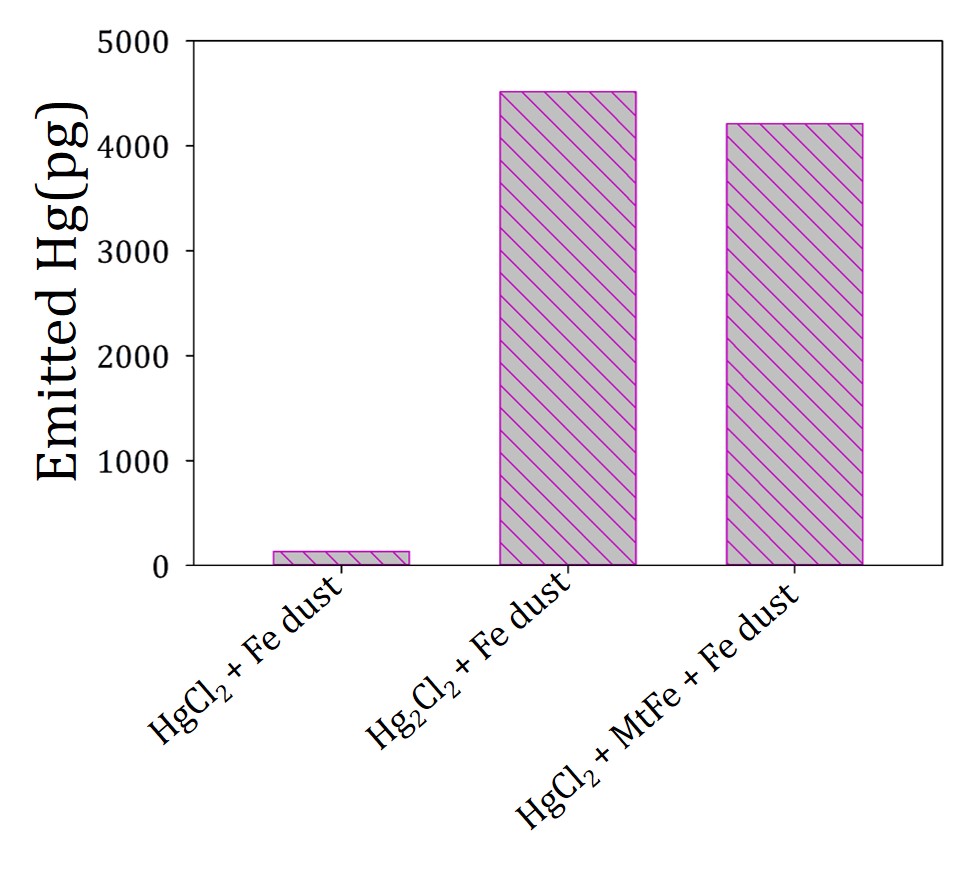


**Figure S12: Emission of elemental Hg from HgCl_2_ (1 mL 10^-2^ M), Hg_2_Cl_2_ and MtFe-Hg (0.0025 g MtFe in 1 mL 10^-2^ M HgCl_2_) with Fe granules of 0.07 g after 16 min. Hg_2_Cl_2_, used, was equivalent mole of HgCl_2_ in 1 mL water.**

**Supporting References**

1. Esmaeili, A., Saremnia, B. & Kalantari. M. Removal of mercury(II) from aqueous solutions by biosorption on the biomass of Sargassum glaucescens and Gracilaria corticata. *Arabian Journal of Chemistry* **8**, 506–511(2015).
2. Krishnan, K. A. & Anirudhan, T. S.  Removal of mercury(II) from aqueous solutions and chlor-alkali industry effluent by steam activated and sulphurised activated carbons prepared from bagassepith: Kinetics And equilibrium studies. *Journal of Hazardous Materials* **92**, 161–183 (2002).
3. Zabihia, M., Ahmadpour, A. & Asl, A. H.  Removal of mercury from water by carbonaceous sorbents derived from walnutshell. *Journal of Hazardous Materials*, **167**, 230–236 (2009).
4. Hassan, S. S. M., [Awaad, N. S. & Aboterika](https://www.sciencedirect.com/science/article/pii/S0304389407016056?via%3Dihub#!) H. A. Removal of mercury(II) from wastewater using camel bone charcoal**.** *Journal of Hazardous Materials* **154**, 992-997 (2008).
5. Ganzagh, M. A. A., Yousefpour, M. &  Taherian, Z. The removal of mercury (II) from water by Ag supported on nanomesoporous silica. *J. Chem. Biol.* **9**, 127–142 (2016).
6. [Zhang](https://www.sciencedirect.com/science/article/pii/S138589471300538X#!), S.,  [Zhang](https://www.sciencedirect.com/science/article/pii/S138589471300538X#!), Y.,  [Liu](https://www.sciencedirect.com/science/article/pii/S138589471300538X#!), J., [Xu](https://www.sciencedirect.com/science/article/pii/S138589471300538X#!), Q.,  [Xiao](https://www.sciencedirect.com/science/article/pii/S138589471300538X#!), H.,  [Wang](https://www.sciencedirect.com/science/article/pii/S138589471300538X#!), X., [Xu](https://www.sciencedirect.com/science/article/pii/S138589471300538X#!), H. &  [Zhou](https://www.sciencedirect.com/science/article/pii/S138589471300538X#!), J. Thiol modified Fe_3_O_4_@SiO_2_ as a robust, high effective, and recycling magnetic sorbent for mercury removal. *Chemical Engineering Journal.* **226**, 30-38 (2013).
7. Saman, N., Johari, K. & Mat H. Adsorption Characteristics of Sulfur-Functionalized Silica Microspheres with Respect to the Removal of Hg(II) from Aqueous Solutions. Ind. Eng. Chem. Res.  **53**, 1225–1233 (2014).
8. Olkhovyk, O., Antochshuk, V. & Jaroniec, M. Thermogravimetric studies of benzoylthiourea-modified MCM-41 after adsorption of mercury ions from aqueous solutions. *Analyst* **130**, 104–108 (2005).
9. Li, C., Huang, S., Min, C., Du, P., Xia, Y., Yang, C. & Huang, Q. Highly Productive Synthesis, Characterization, and Fluorescence and Heavy Metal Ion Adsorption Properties of Poly(2,5-dimercapto-1,3,4- thiadiazole) Nanosheets. *Polymers* **10**, 24 (2018).
10. Luan, Z., Fournier, J. A., Wooten, J. B., Miser, D. E. & Chang, M. J. Functionalized mesoporous SBA-15 silica molecular sieves with mercaptopropyl groups: Preparation, characterization and application as adsorbents. *Studies in Surface Science and Catalysis* **156**, 897-906 (2005).
11. Prado, A. G. S., Arakaki, L. N. H. & Airoldi, C. Adsorption and separation of cations on chemically modified silica gel synthesised via the sol-gel process. *J. Chem. Soc. Dalton Trans.* **0**, 2206–2209 (2001).
12. Song, J., Kong, H. & Jang, J. Adsorption of heavy metal ions from aqueous solution by polyrhodanine-encapsulated magnetic nanoparticles. *J. Colloid Interface Sci.* **359**, 505–511 (2011).
13. Lee, B., Kim, Y., Lee, H. & Yi, J. Synthesis of functionalized porous silicas via templating method as heavy metal ion adsorbents: The introduction of surface hydrophilicity onto the surface of adsorbents. *Microporous Mesoporous Mater.* **50**, 77–90 (2001).
14. Shin, S. & Jang, J. Thiol containing polymer encapsulated magnetic nanoparticles as reusable and efficiently separable adsorbent for heavy metal ions. *Chem. Commun.* **2007**, 4230–4232 (2007).
15. Tao, S., Wang, C., Ma, W., Wu, S. & Meng, C. Designed multifunctionalized magnetic mesoporous microsphere for sequential sorption of organic and inorganic pollutants. *Microporous Mesoporous Mater.* **147**, 295–301 (2012).
16. Li, B., Zhang, Y., Ma, D., Shi, Z. & Ma, S. Mercury nano-trap for effective and efficient removal of mercury(II) from aqueous solution. *Nat. Commun.* **5**, 5537 (2014).
17. Shin, Y. *et al.* Sulfur-functionalized mesoporous carbon. *Adv. Funct. Mater.* **17**, 2897–2901 (2007)
18. Bag, S., Trikalitis, P. N., Chupas, P. J., Armatas, G. S. & Kanatzidis, M. G. Porous semiconducting gels and aerogels from chalcogenide clusters. *Science* **317**, 490–493 (2007)
19. Yee, K. -K. *et al.* Effective mercury sorption by thiol-laced metal−organic frameworks: in strong acid and the vapor phase. *J. Am. Chem. Soc.* **135**, 7795–7798 (2013)
20. Wahi, R., Ngaini, Z. & Jok, V. U. Removal of Mercury, Lead and Copper from Aqueous Solution by Activated Carbon of Palm Oil Empty Fruit Bunch. *World Applied Sciences* *Journal* **5**, 84-91 (2009).
21. Zhai, Q. –Z. Nano α-Al2O3 for removal of Hg(II) from water: Adsorption and desorption studies. *Journal of Chemical and Pharmaceutical Research* **6**, 1310-1317 (2014).
22. Meena, A. K., Mishra, G. K., Kumar, S. & Rajagopal, C. Low-cost Adsorbents for the Removal of Mercury (11) from Aqueous Solution-A Comparative Study*. Defence Science Journal* **54**, 537-548 (2004).
23. Razaee, A., Derayat, J., Godini, H. & Pourtaghi, G. Adsorption of mercury from synthetic solutions by an Acetobactor xylinum biofilm. *Res. J. Environ. Sci.* **2**, 401-407 (2008).
24. E I El-Shafey, C., Pichugin, A.  A. & Appleton, Q. Removal of mercury(II) from aqueous solution on a carbonaceous sorbent prepared from flax shive. *J. Chem. Technol. Biotechnol.***75**, 427–435 (2000).
25. Mergola, L., Scorrano, S., Bloise, E., Bello, M. P. D., Catalano, M., Vasapollo, G. & Sole, R. D. Novel polymeric sorbents based on imprinted Hg(II)-diphenylcarbazone complexes for mercury removal from drinking water. **48**, 73-79 (2016).
26. Bhattacharyya, K. G. & Gupta, S. S. Adsorption of a few heavy metals on natural and modified kaolinite and montmorillonite: A review. *Advances in Colloid and Interface Science* **140**, 114–131 (2008).
27. Sangu, V., Kannan, K. & Srinivasan, K. Removal of mercury (II) ion from the aqueous solution using a synthetic terpolymer. *Ind. J. Chem. Tech.* **22**, 219-226 (2015).
28. Rahbar, N., Jahangiri, A., Boumi, S. & Khodayar, M. J. Mercury Removal From Aqueous Solutions With Chitosan-Coated Magnetite Nanoparticles Optimized Using the Box-Behnken Design. *Jundishapur J. Nat. Pharm. Prod.* **9**, e15913 (2014).
29. Lalhmunsiama, Lee, S. M., Choi, S. S. & Tiwari, D. Simultaneous Removal of Hg(II) and Phenol Using Functionalized Activated Carbon Derived from Areca Nut Waste. *Metals* **7**, 248 (2017).
30. Baheti, V., Vellora, V., Padil, T., Militky, J., Cernik, M. & Mishra, R. Removal of Mercury from Aqueous Environment by Jute Nanofiber. *Journal of Fiber Bioengineering and Informatics* 6, 175–184 (2013).
31. Tuzen, M., Sari, A., Mendil, D., Soylak, M. Biosorptive removal of mercury (II) from aqueous solution using lichen (Xanthoparmelia conspersa) biomass: Kinetic and equilibrium studies. *J. Hazard. Mater.* **169**, 263-270 (2009).
32. Bayramoˇglu, G.; Tuzun, I.; Celik, G.; Yilmaz, M., Arica. Y. Biosorption of mercury (II), cadmium (II) and lead (II) ions from aqueous system by micro algae Chlamydomonas reinhardtii immobilized in alginate beads. *Int. J. Miner. Process* **81**, 35-43 (2006).
33. Vinod, V. T. P., Sashidhar, R. B., Sivaprasad, N., Sarma, U. V. M., Satyanarayana, N., Kumaresan, R., Rao, T. N. & Raviprasad P. Bioremediation of mercury (II) from aqueous solution by gum karaya (Sterculia urens): A natural hydrocolloid. *Desalination* **272**, 270-277 (2011).
34. Barriada, J. L., Herrero, R., Rodrguez, D. P. & Sastre de Vicente, M. E. Interaction of mercury with chitin: A physicochemical study of metal binding by a natural biopolymer. *React. Funct. Polym.* **68**, 1609-1618 (2008).
35. Edelio T., Cardenas G. & Orlando P. Synthesis and applications of chitosan mercaptanes as heavy metal retention agent. *Int. J. Biol. Macromol* **28**, 167-174 (2001).
36. Meena, A. K., Kadirvelu, K., Mishra, G. K., Rajagopal, C. & Nagar, P. N. Adsorptive removal of heavy metals from aqueous solution by treated sawdust (Acacia arabica). *J. Hazard. Mater.* **150**, 604-611 (2008).
37. Zhu, H., Shen, Y., Wang, Q., Chen, K., Wang, X., Zhang, G., Yang, J., Guo, Y. & Bai, R. Highly promoted removal of Hg(II) with magnetic CoFe2O4@SiO2 core–shell nanoparticles modified by thiol groups. *RSC Adv.* **7**, 39204–39215 (2017).
38. Liu, T., Wang, Z. –L., Yan, X. & Zhang, B. Removal of mercury (II) and chromium (VI) from wastewater using a new and effective composite: Pumice-supported nanoscale zero-valent iron. *Chemical Engineering Journal* **245**, 34–40 (2014).

**Table S1: Removal of Hg(II) in spiked water samples**

| Sample | Conc. of Hg (mg L^-1^) | Removal (%) |
| --- | --- | --- |
| Tap water (McGill University) | 2000  1000  500  100 | 96%  99%  94%  96% |
| Saint Laurent river (Montreal, Canada) | 2000  1000  500  100 | 92%  98%  93%  95% |
| Melted snow (Burnside building, McGill University) | 2000  1000  500  100 | 99%  94%  94%  97% |
| Rain water (Montreal) | 2000  1000  500  100 | 94%  98%  92%  97% |
